# Frequency of acute longus colli tendinitis on CT examinations

**DOI:** 10.1007/s10140-017-1537-z

**Published:** 2017-07-25

**Authors:** John Boardman, Emanuel Kanal, Patrick Aldred, Joseph Boonsiri, Chijindu Nworgu, Feng Zhang

**Affiliations:** 1 Radiology Suite, 201 East Wing, 200 Lothrop St, Pittsburgh, PA 15213 USA; 2 Radiology Suite, 200 Lothrop St, Pittsburgh, PA 15213 USA; 3 Department of Neuroradiology, NYU, 660 First Ave., 2nd Floor, New York, NY 10016 USA; 4 Department of Radiology Education, Stanford University Medical Center, 300 Pasteur Dr., H1330, MC:5621, Stanford, CA 94305-5621 USA

**Keywords:** Longus colli, Retropharyngeal tendinitis, Prevertebral tendinitis

## Abstract

**Purpose:**

We attempted to determine the frequency of acute longus colli tendinitis on diagnostic CT imaging performed at a large multicenter health care system. By correlating with the pre-imaging clinical information, we investigated which patient presentations should lead the radiologist to increased suspicion for this condition.

**Methods:**

Images from a total of 8101 adult CT examinations of the neck and cervical spine performed over a 3-month period were evaluated by researchers independent of the original clinical report. Clinical information available at the time of imaging was reviewed and assigned to one of five categories. Frequency of the condition was calculated by sex and clinical presentation. This retrospective study with waiver of consent and waiver of HIPPA was approved by our IRB.

**Results:**

Nine positive scans were found for an overall frequency of 1.1 per 1000 examinations. The frequency was significantly higher (11.4 per thousand) on scans performed of patients presenting without history of recent trauma, concern for tumor, suspected postoperative complication, or clinical signs of infection localized to the neck. Although frequency in males was higher than in females, this did not reach statistical significance. In no positive or negative case was longus colli tendinitis considered in the pre-imaging documentation.

**Conclusions:**

Findings of acute longus colli tendinitis on CT examination generally occur in the absence of prior mention of this condition in the medical record. The radiologist should be particularly alert for this diagnosis when a patient presents with rapid-onset neck pain without a clear history of recent trauma or other etiologies.

## Introduction

Longus colli tendinitis first appeared in an English language publication as a case report in 1964 [1]. A 52-year-old male then presented with several days of worsening neck pain with associated muscle spasm, odynophagia, and globus sensation. He denied recent trauma. A lateral radiograph of the cervical spine revealed an area of calcific density anterior to the vertebral column at C1 and C2. The patient responded well to anti-inflammatory medication, and the calcific density resolved on successive plain film examinations.

In succeeding years, the condition was reported in the radiology and other medical literature as a distinct etiology for acute non-traumatic neck pain. Its plain film appearance included both calcific deposits anterior to the spine at the C1 and C2 levels as well as thickening of soft tissues anterior to the mid and upper cervical vertebral column. Acute longus colli tendinitis was first described on CT examination in 1986 [2]. On CT, the calcification is seen to lie at the upper myotendinous junction of the superior oblique fibers of the longus colli muscle at the C1–2 level [3]. It displays no evidence of cortication or internal trabecula, and the appearance is described by multiple authors as “amorphous” [2–5]. Bilaterally symmetric edema originates in the prevertebral space with variable extension into the retropharyngeal space in the upper and mid neck [6]. On contrasted images, no enhancement is seen in association with this edema [4, 7, 8]. Elevated white count is present in a substantial number of patients and low-grade fever in up to 35% of patients [7–10].

CT examination has come to be recognized as significantly more sensitive than plain radiographs for both the calcification and the edema of this condition. The presence of both these findings on CT is considered highly characteristic for acute longus colli tendinitis [6, 7, 10, 11]. Resolution of both calcification and edema has been observed on both follow-up plain film and CT examinations [1, 5, 9, 12].

Biopsy of the calcific deposits has demonstrated intratendinous calcium granuloma lesions containing heterogeneous plate-shaped crystals of calcium hydroxyapatite [3]. This condition is now considered to represent another member of the family of calcium hydroxyapatite deposition diseases, along with more common manifestations at the shoulder, hips, and other joints [11, 13]. For this reason, we prefer to use the term longus colli tendinitis. However, multiple variant names are found in the literature, including calcific prevertebral tendinitis, prevertebral calcific tendinitis, calcific retropharyngeal tendinitis, and retropharyngeal calcific tendinitis.

With the widespread adoption of cervical spine or neck CT as a screening examination in emergency departments, awareness of the CT findings of this condition has become increasingly important. The retropharyngeal edema may appear very prominent on CT and suggest an infectious etiology. However, the simultaneous finding of the characteristic calcification with symmetric edema should distinguish acute longus colli tendinitis from infection [8, 11, 14]. Although acute longus colli tendinitis responds readily to anti-inflammatory medications, particularly nonsteroidal anti-inflammatory drugs (NSAIDs), there are reports of extensive workups for infectious disease, neoplasm, and even surgical excision of what was believed preoperatively to be a calcified tumor [3].

During the early autumn of 2014, multiple radiologists at our institution anecdotally noticed a large number of cases on CT examinations of the neck region. A literature search revealed no statistically valid investigation of the frequency of acute longus colli tendinitis on CT examinations. Existing case series reported patients ranging in age from 21 to 81 years without consistent male or female predominance [3, 8, 15, 16]. The presenting symptoms were most prominently neck pain, dysphagia, and odynophagia in the absence of trauma, although a limited number of patients reported recent trauma as a possible etiology [3, 16].

We decided to perform a review of all neck and cervical spine CT examinations performed over a 3-month interval to determine the overall frequency of this condition as well as possible variation with age, sex, and clinical presentation.

## Methods

A prospective study period of November 1, 2014, through January 31, 2015, was chosen. A retrospective review was made of all CT imaging studies of the neck region performed in this period. These included both cervical spine CT examinations and neck soft tissue examinations, performed either with or without intravenous contrast. Examinations performed as part of interventional procedures (CT-guided discography, nerve root block, and facet injections) were excluded. Facilities covered included three academic medical centers, a women’s hospital, a pediatric hospital, and 11 community hospitals. Because this study included diverse institutions ranging from university-affiliated academic medical centers to small community hospitals, there was a considerable variation of protocols. Where the examination was performed as a cervical spine CT, axial bone images were provided at 1.25-mm intervals and, in many instances, at 0.62-mm intervals. Most institutions also provided additional axial images in soft tissue or standard algorithm. Sagittal and coronal reformats were available in bone algorithm and sometimes in soft tissue or standard algorithm. Where the examination was performed as a neck soft tissue CT, soft axial images were performed at thickness of 2.5 mm or less, with sagittal and coronal reformats. Where intravenous contrast was administered, the dose ranged from 60 to 100 ml of Iopamidol (Isovue) 370.

The age, sex, and presenting symptoms of all patients were tabulated. Hematologic laboratory results and temperature on presentation were recorded when available. The post-imaging clinical course of those patients considered positive was noted when available. Approval of this study with waiver of patient consent and waiver of HIPPA was obtained from our institutional review board.

The principal clinical indication for each examination was assigned to one of the five following categories:

1. Trauma occurring within the past 14 days. This time period was chosen empirically, based on the observation that clinicians generally describe trauma occurring more than 14 days previous as “remote.”
2. Evaluation for neoplasm, including neck mass palpable on physical examination, previously diagnosed tumor of the neck or cervical spine, or concern for metastatic spread of known primary tumor at another anatomic location.
3. Evaluation for suspected postsurgical complication including hematoma, abscess, perforation of the esophagus or trachea, or hardware failure.
4. Clinical signs concerning for infection localized to the neck, including purulent drainage from a sinus tract, enlarged and inflamed tonsils, dental caries with jaw swelling, ear pain with mastoid tenderness, and swollen salivary glands concerning for sialadenitis. Elevated white count and/or fever was not of itself sufficient for assignment to this category.
5. All other indications, including neck pain in the absence of recent trauma, dysphagia, odynophagia, and globus sensation.

Assignment was made on the basis of clinical information available prior to interpretation of the study. For example, if a scan performed to evaluate for possible failure of fixation hardware revealed an incidental laryngeal mass, the study was still assigned to category 3.

Images were analyzed for the presence of amorphous calcification near the upper insertion of the longus colli muscle and of edema in the prevertebral or retropharyngeal space. An examination was considered positive only if both these findings were present. An initial review was made by the four co-authors then in our diagnostic radiology residency program. All studies were subsequently reviewed by the first author of this study, a CAQ-qualified staff neuroradiologist with 12 years of experience. It was agreed that the first author would make the final decision on which studies were positive. Edema was quantified as follows: the first author measured anteroposterior extent of edema using the “ruler” function of our Phillips ISite PACS system. Measurements were taken in anteroposterior direction on the several axial images where edema appeared to achieve its greatest thickness. The largest such measurement was rounded to the nearest millimeter and recorded as maximum thickness. Craniocaudad extent was determined by recording the highest and lowest axial images with convincing appearance of edema and then subtracting the table positions of these two slices.

The age distribution of positive and negative cases was compared using the Mann-Whitney test, both for all patients and for males and females considered separately. Frequency of positive cases per 1000 exams was computed for the entire patient population and for subsets by sex and clinical presentation. The significance of variation in frequency by sex and over different clinical presentations was evaluated using the chi-square test.

## Results

A total of 8416 CT examinations of the neck or cervical spine were performed during the study period, including 315 patients less than 18 years of age. Because no pediatric case was positive in this study and because the youngest case recorded in the literature was aged 21 years [15], it was decided to restrict statistical analysis to patients 18 years or older at the time of examination. Five thousand six hundred sixty-three adults were imaged while in an emergency department, 1979 as outpatients and 459 as inpatients. One thousand eight hundred seventy-one examinations were performed with intravenous contrast and 6230 without. There were 7 positive cases (aged 38 to 59) out of 3749 examinations performed in adult male patients (aged 18 to 102) (Table 1). Two positive cases (aged 63 and 82) were found on 4352 examinations of adult females (aged 18 to 107 years). Both the characteristic calcification and symmetric edema were unambiguous in all positive cases, and no discordances arose between researchers. In all positive cases, no plausible etiology other than longus colli tendinitis could be found for the patient’s symptoms. The age of positive examinations did not differ significantly from that of negative examinations when males and females were grouped together (*P* = .53) or for males (*P* = .26) and females (*P* = .44) considered separately.

**Table 1:** Adult patient age and sex distribution

	All examinations	Positive examinations
Male		
*N* total	3749	7
*N* with contrast	934	3
Age range (years)	18 to 102	38 to 59
Mean ± SD	56.2 ± 19.9	50.0 ± 9.2
Female		
*N* total	4352	2
*N* with contrast	937	0
Age range (years)	18 to 107	63 to 82
Mean ± SD	60.6 ± 21.5	72.5 ± 13.4
Total		
*N* total	8101	9
*N* with contrast	1871	3
Age range (years)	18 to 107	38 to 82
Mean ± SD	58.6 ± 20.9	55.0 ± 13.6

**Fig. 1:**
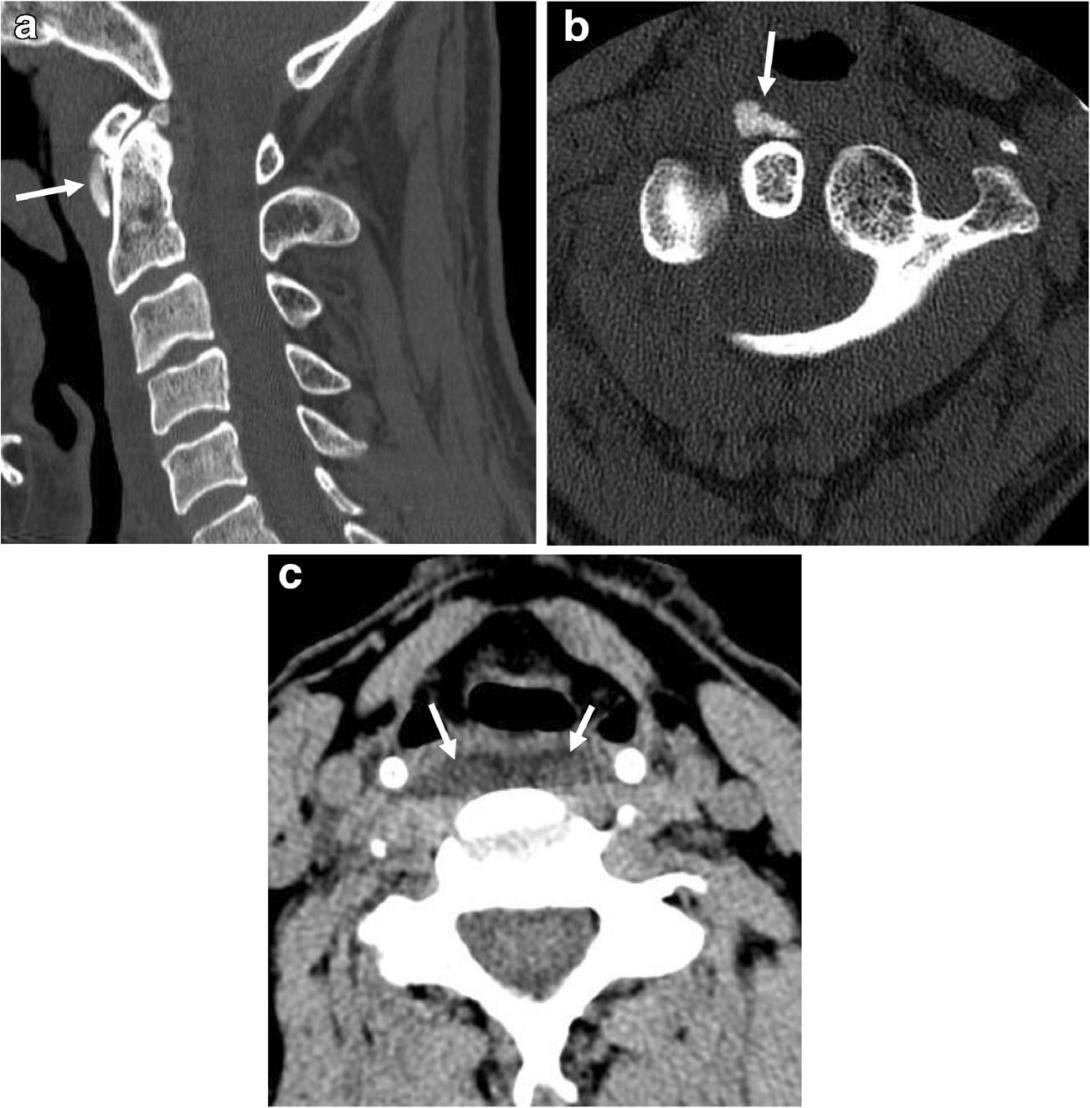
Cervical spine CT examination performed on a 59-year-old male (patient 6) presenting with neck pain and subjective fever without recent trauma. Sagittal (**a**) and axial (**b**) images show the characteristic amorphous calcification (*arrows*) of longus colli tendinitis at the upper myotendinous junction of this muscle. Axial image (**c**) demonstrates symmetric edema (*arrows*) outlining the anterior margin of the longus colli muscles

The calcification in all positive cases demonstrated the previously described amorphous appearance (Fig. 1a, b). The associated edema measured between 3 and 8 mm in the greatest anteroposterior dimension and between 18 and 55 mm in the craniocaudad extent. In all cases, edema was symmetric and outlined the anterior margin of the bilateral longus colli muscles (Fig. 1c). In the three positive studies performed with intravenous contrast (Table 2, patients 1, 3, and 5), there was no perceptible enhancement associated with the edema.

**Table 2:** Characteristics of positive patients

Patient	Sex	Age	Setting	Presenting symptoms	Temp.	WBC
1	M	38	ED	2 days of neck pain increased with motion, pain with swallowing, and chewing	36.7	8.4
2	M	39	ED	10 days of neck and upper back pain	36.8	10.5
3	M	46	ED	2 days of neck pain and sore throat progressing to difficulty in swallowing	36.4	20.4
4	M	51	ED	“Achy” neck pain after fall on carpeted stairs 1 day previous	36.7	NA
5	M	58	ED	3 days of neck pain, worsened with motion or swallowing	36.5	9.0
6	M	59	ED	2 weeks of neck pain and subjective fever	36.8	10.9
7	M	59	Outpatient	Headache, neck pain, and dysphagia 2 days post minor motor vehicle trauma	NA	NA
8	F	63	Outpatient	5 days of pain in the posterior and right side of neck, shooting pain into head, difficulty in swallowing	NA	NA
9	F	82	ED	2 weeks of neck and back pain, and weakness	36.7	6.7

*NA* not available

The reported duration of symptoms in positive patients ranged from 2 days to 2 weeks (Table 2). All positive patients complained of neck pain. Five patients experienced either pain or difficulty in swallowing. No patient specifically mentioned globus sensation. Seven patients denied recent trauma, and two male patients experienced neck pain in the setting of recent trauma. Patient 4 complained of “achy” neck pain 1 day after a fall on carpeted stairs. Patient 7 presented with headache, neck pain, and dysphagia 2 days after minor motor vehicle trauma.

Hematology laboratory results at the time of presentation are available for six of the positive patients. Two individuals (patients 2 and 6) presented with minimally elevated white count of 10,500 and 10,900 total white cells per microliter, respectively. Both were afebrile and showed no clinical signs of infection. Patient 3 presented with marked leukocytosis (20,400 total white cells/μl) and neutrophilia (17,900/μl, the upper limit of normal at our laboratory is 7500). His temperature on presentation was 36.4 °C. Flexible laryngoscopy showed a small amount of posterior hypopharyngeal wall swelling with no evidence of exudate or abscess. Both sputum and blood cultures remained negative. His pain and difficulty in swallowing improved dramatically after overnight treatment with intravenous steroids. He was afebrile on discharge, and repeat white count was not obtained. With the exception of patient 7, who was lost to follow-up, all patients recovered with conservative treatment.

The frequency of acute longus colli tendinitis varied markedly between the various clinical presentations (Table 3). It was 1.1 ± 0.6 per thousand when calculated over all examinations performed, but reached 11.4 ± 7.1 per thousand when limited to examinations performed for the “other” category of clinical presentation. Variation of frequency over the five clinical indications was highly significant (*P* < .001) when analyzed for male and female patients separately, as well as for all patients together. Data was also analyzed by combining the first four clinical indications as a “specific” category and comparing with the fifth “other” category. There was a highly significant variation in frequency between “specific” and “other” indications (*P* < .001) both for males and females considered separately and for all patients grouped together. Although more male than female patients were positive in this study, the gender difference did not reach significance either among all nine patients (*P* = .06) or when analysis was confined to the seven patients in the “other” category (*P* = .24).

**Table 3:** Distribution by sex and presentation

	Trauma	Neoplasm	Surgery	Infection	All specific presentations	Other presentations	All presentations
Male							
*E*	2361	815	153	117	3446	303	3749
*P*	2	0	0	0	2	5	7
*F*	0.8 ± 1.0	0	0	0	0.6 ± 0.7	16.5 ± 12.0	1.9 ± 1.2
Female							
*E*	2930	841	151	119	4041	311	4352
*P*	0	0	0	0	0	2	2
*F*	0	0	0	0	0	6.4 ± 7.5	0.5 ± 0.5
Total							
*E*	5291	1656	304	236	7487	614	8101
*P*	2	0	0	0	2	7	9
*F*	0.4 ± 0.4	0	0	0	0.3 ± 0.3	11.4 ± 7.1	1.1 ± 0.6

*E* = total examinations performed, *P* = the number of positive examinations, *F* = frequency per thousand examinations ± 90% confidence interval

## Discussion

We believe that no prior research exists investigating the frequency of acute longus colli tendinitis on a large sample of CT examinations. A 2014 study from Chile was conducted by retrospectively text searching clinical reports of CT neck examinations performed with intravenous contrast at a Santiago hospital [17]. Only studies whose existing diagnostic radiology reports included specific terms related to longus colli tendinitis were considered to meet inclusion criteria. Because longus colli tendinitis is an often overlooked diagnosis, it is possible that some positive cases were not recorded in the text reports searched. In addition, cervical spine and neck CT examinations performed without intravenous contrast were not searched.

A 2013 study performed in Tel Aviv found eight positive cases out of 13 CT examinations ordered specifically to evaluate for longus colli tendinitis [18]. However, these 13 studies were performed only after thorough clinical examination (including fiber-optic endoscopy) by an otolaryngologist raised a high clinical suspicion for longus colli tendinitis. No attempt was made to evaluate frequency of the condition on examinations ordered by emergency physicians or generalist physicians.

The largest published case series comes from Denmark in 2009 [19]. By querying multiple chiropractic clinics, the authors retrospectively identified 45 cases. Unfortunately, it is unclear which patients were imaged with plain radiographs and which with CT. The authors did not determine how many imaging examinations were performed to yield 45 positives.

In the current study, seven of nine positive patients presented with the commonly reported symptoms of non-traumatic neck pain, dysphagia, or odynophagia. The etiology of tendinitis in the two patients with recent history of trauma is unclear. Patient 4 did not summon paramedics after a fall on carpeted stairs. However, when he experienced continuing “achy” neck pain, he transported himself to an emergency department the next day. Patient 7 had an outpatient cervical spine CT ordered by his physician 2 days after minor motor vehicle trauma. Occurrence of longus colli tendinitis shortly after trauma has been previously noted in two patients reported in a 2012 case series from Korea. [16]. In that series, one patient presented 2 days after a fall and another 1 day after hyperextension to the neck caused by walking into a ladder. It has been established by biopsy that the calcific density observed in longus colli tendinitis represents hydroxyapatite crystal deposition [3]. The calcific deposits were unlikely to result from trauma, given the short period between injury and presentation. It has been posited that the acute symptoms of longus colli tendinitis arise when pre-existing crystals rupture and induce a severe inflammatory reaction [4, 20]. Conceivably, patients 4 and 7 had such hydroxyapatite deposits in their longus colli tendon, which ruptured in response to relatively minor trauma. We agree with the authors of the Korean study that to exclude this diagnosis in the presence of recent trauma would risk missing a significant number of cases.

The lack of enhancement on the three positive studies performed with intravenous contrast during the current research confirms multiple prior case reports. We conclude that contrast is not needed to make this diagnosis and can be omitted without reducing the sensitivity of CT examination.

We believe that the current study avoids the statistical flaws of case series that only evaluate already known positive cases. By reviewing the actual archived images independent of the contemporaneous diagnostic interpretation and with specific attention to longus colli tendinitis, we should achieve optimum sensitivity and specificity, not limited by the receiver operating characteristics of multiple clinical radiologists. We are also aware that where a condition is rare, normal statistical fluctuations may give a falsely high or low estimate of its occurrence. For this reason, a prospective study period was determined in advance, rather than including the time period where an apparently unusual number of cases caught the attention of the authors. Because the study was limited to examinations performed in a fairly compact area during a 3-month period, we may be missing geographic and seasonal variations in frequency. In addition, longus colli tendinitis is generally a self-limiting condition that often escapes medical attention, and therefore, we are unable to make any estimate of a population-based incidence.

At the authors’ multicenter institution, neck pain accounts for a large portion of the CT examinations performed. The frequency of acute longus colli tendinitis was found to be truly low, 1.1 ± 0.6 (90% confidence interval) per thousand when computed over all neck and cervical spine CT examinations performed. However, this rises to 11.4 ± 7.1 per thousand when limited to patients without history of recent trauma, tumor, concern for postoperative complications, or clinical signs of infection localized to the neck. Although confidence intervals are wide, we believe that the current study produces credible evidence establishing frequency on CT examination to well within the order of magnitude accuracy.

An informative comparison can be made with examinations ordered for headache. A large (*N* = 3028) meta-analysis of head CT examinations ordered for both acute and chronic headache [21] found evidence of brain tumor on only 8 per thousand examinations. In the current study, we found that where CT imaging of the neck was ordered in the absence of trauma, suspicion for neoplasm, concern for surgical complication, or signs of infection localized to the neck, the frequency of longus acute colli tendinitis was 11.4 per thousand. Every competent radiologist includes tumor in the search pattern for evaluating head CTs. Unfortunately, longus colli tendinitis may be overlooked or misinterpreted by clinical radiologists. Failure to recognize the findings of this benign condition can lead to needless and possibly invasive workups.
